# Magnetic Microparticles With Immobilized Bovine Serum Albumin as a Stationary Phase for Affinity Studies Using Pressure‐Assisted Capillary Electrochromatography. Proof of Concept

**DOI:** 10.1002/jssc.70570

**Published:** 2026-11-09

**Authors:** Michal Sedlák, Lenka Kohútová, Marta Pelcová, Zdeněk Glatz

**Affiliations:** ^1^ Department of Biochemistry Faculty of Science Masaryk University Brno Czech Republic

**Keywords:** binding parameters, drug, magnetic particles, pressure‐assisted capillary electrochromatography, serum albumin

## Abstract

This study presents a novel pressure‐assisted capillary electrochromatography method that uses a fritless capillary column of magnetic microparticles with immobilized serum albumin to investigate the affinity interactions of drugs with plasma proteins. To the authors' knowledge, this is the first time that this type of column has been used in a study examining the binding properties of a drug–serum albumin pair. The use of magnetic particles as the chromatographic bed material offers several advantages, such as easy handling during chemical activation and subsequent protein immobilization, and relatively simple column preparation achieved by external permanent magnets around the capillary. In addition, this concept is characterized by low protein and reagent consumption, which is typical for capillary electrochromatography. Glutaraldehyde coupling was chosen for its sufficient yield of immobilized bovine serum albumin (BSA) with preserved drug‐binding properties compared to the carbodiimide method. Furthermore, the required binding capacity was increased by optimizing the length of the particle bed, which was held in the capillary by 25 pairs of permanent magnets in an attractive orientation in a 3D‐printed holder positioned inside the capillary cartridge. After optimizing the immobilization procedure and the column preparation, the column was used to determine the binding parameters of the model drug phenylbutazone (PBZ) to the pharmacologically significant Sudlow I site on the BSA molecule. Frontal electrokinetic injection and breakthrough time analysis were employed for each PBZ concentration to estimate the corresponding equilibrium binding constant. The proposed approach could be particularly useful for studying the binding of drugs to expensive or rare proteins and support the choice of lead compounds in drug development studies.

## Graphical abstract

This study investigated the utility of capillary electrochromatography columns based on magnetic particles with immobilized serum albumin for studying the binding of drugs to plasma proteins. To the best of the authors’ knowledge, this is the first report describing an affinity interaction between drugs and bovine serum albumin using this type of column.

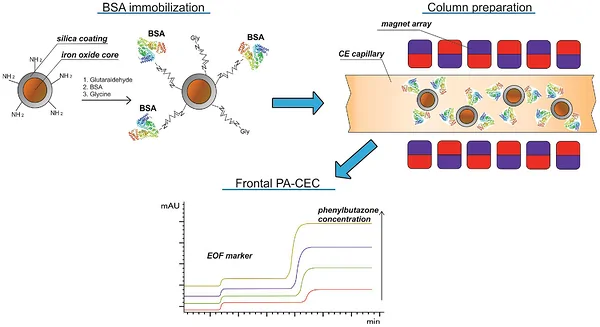

**Abbreviations**
ACE: affinity capillary electrophoresis
ACEC: affinity capillary electrochromatography
BCA: bicinchoninic acid assay
BGE: background electrolyte
BSA: bovine serum albumin
CE‐FA: capillary electrophoresis—frontal analysis
DW: deionized water
EDC: 1‐ethyl‐3‐(3‐dimethylaminopropyl)carbodiimide
EOF: electroosmotic flow
FA: frontal analysis
GA: glutaraldehyde
HEPES: 2‐[4‐(2‐hydroxyethyl)piperazin‐1‐yl]ethane‐1‐sulfonic acid
HPAC: high‐performance affinity chromatography
HSA: human serum albumin
IB: incubation buffer
MES: 2‐(N‐morpholino)ethanesulfonic acid
msACE: mobility shift affinity capillary electrophoresis
OT: open tubular
OT‐CEC: open tubular capillary electrochromatography
PA‐CEC: pressure‐assisted capillary electrochromatography
PB: phosphate buffer
PBZ: phenylbutazone
RSD: relative standard deviation
SEI: short‐end injection
S‐NHS: sulfo‐N‐hydroxysuccinimide

## 1 Introduction

A variety of pharmacokinetic processes, including drug liberation, absorption, distribution, metabolism, elimination, and toxicology—LADMET, are significantly influenced by their binding to plasma proteins [1, 2]. This is due to the fact that these proteins, particularly human serum albumin (HSA), which is the most abundant, act as reservoirs and also mediate drug transport [3]. In this study, bovine serum albumin (BSA), a protein that is structurally and functionally very similar to HSA [4], was used instead due to its easier accessibility. According to the free drug theory, the unbound drug concentration in plasma equals that at the tissue target site under passive steady‐state conditions [5]. Consequently, any factor altering unbound drug concentrations directly modulates the therapeutic effect. Additionally, the potential for plasma protein binding displacement to act as a mechanism of drug‐drug interactions during multidrug therapy should be considered [6]. As a result, a comprehensive understanding of the mechanism of interaction requires the determination of pivotal parameters such as the equilibrium binding constant (K_b_) and the stoichiometry of the interaction, including identifying the drug‐binding sites in the structure of plasma proteins. It is therefore imperative that these interactions are characterized in the early phases of new drug development, where thousands of leads have to be tested, so high‐throughput screening technologies are required.

A wide range of techniques, differing in their basic principle, have been developed over time to characterize the above‐mentioned interactions. The first of these techniques was equilibrium dialysis, which was applied by Waddell et al. in 1957 to investigate the interactions between HSA and phenobarbital, and was subsequently established as the gold standard for this purpose [7]. In 1969, Rehse conducted an HSA‐drug analysis using nuclear magnetic resonance spectroscopy [8]. In the same year, Chignell used a circular dichroism method to estimate the equilibrium binding constant of the HSA‐phenylbutazone (HSA‐PBZ) pair [9], and in 1970, he employed a fluorescence‐based technique to study HSA‐warfarin binding [10]. Subsequently, in 1978, the interactions of HSA with warfarin and furosemide were measured by high‐performance liquid chromatography [11] and, in 1979, Manzini et al. used a microcalorimetric technique to study HSA‐drug interactions [12]. Several studies on the application of continuous ultrafiltration to investigate HSA‐warfarin interactions were published in the 1980s [13]. Additionally, in 1990, Herrera et al. employed a microdialysis method to characterize HSA‐drug binding. In 1992, Kraak et al. studied HSA‐drug binding parameters using a capillary electrophoresis‐frontal analysis (CE‐FA) method [14]. Another highly efficient method for investigating HSA‐drug interactions is surface plasmon resonance, as demonstrated by Frostell‐Karlsson et al. in 2000 [15]. Finally, it is also worth mentioning the growing interest in using computational chemical methods to elucidate the role of protein binding in a drug's action. In 2005, Hanai used this method to predict the equilibrium binding constant of acidic drugs to HSA [16].

In addition to CE‐FA, many other affinity‐based CE (ACE) experimental approaches have been developed to study biomolecular interactions, including those between plasma proteins and drugs. These approaches are based either on changes in the mobility of the interaction partners after association, with mobility shift ACE (msACE) being the most prevalent, or on determining the amounts of free and bound partners, as in CE‐FA [17, 18]. These two methods, msACE and CE‐FA, are the most utilized ACE variants for characterizing plasma protein‐drug binding. In addition to the classic CE benefits of high separation efficiency, minimal sample consumption, high throughput, and limited sample pretreatment, they offer several other advantages, such as the elimination of the need for highly purified samples, immobilization, and labelling of any of the interacting partners.

On the other hand, another capillary‐based electromigration method—capillary electrochromatography (CEC) uses one interaction partner bound to a solid support. This hybrid method utilizes one of the basic electrokinetic phenomena of capillary electromigration methods—electroosmotic flow (EOF)—to drive the mobile phase/background electrolyte (BGE) through the separation capillary/column, as well as the principle of chromatography, which involves the distribution of analytes between the mobile and stationary phases. This combination enables the separation of analytes based on their mobility in the electric field and their interaction with the stationary phase [19]. The flat EOF profile of CEC ensures a higher separation efficiency than pressure‐driven liquid chromatography with a parabolic profile of hydrodynamic flow, while retaining the aforementioned advantages of CE. Since its introduction, CEC has been used to separate and analyze a wide range of low‐ and high‐molecular‐mass compounds, including studies of biomolecular interactions. This is particularly true for chiral separations, which, depending on the chiral selector used, can also be considered to be based on biomolecular interactions [20, 21].

In CEC, or more precisely in affinity CEC (ACEC), one interaction partner is thus immobilized onto the particle or monolithic‐based stationary phase, or directly onto the capillary wall. In this manuscript, magnetic particles with immobilized BSA and a magnetic field formed by external magnets were used to prepare packed columns for an ACEC study of plasma protein–drug affinity interactions. It should be mentioned that BSA, a stable and inexpensive variant of serum albumin, is frequently used as a model protein to explore binding interactions [22].

Magnetic particles and an external magnetic field were first used in CEC by Oguri et al., who used them to create frits for particle‐based stationary phase immobilization in packed CEC [23]. In 2007, Wang et al. prepared the first fritless packed CEC column using magnetic octadecylsilane‐bound silica particles [24]. Zhu et al. introduced a new approach for preparing open‐tubular (OT) CEC columns by attaching magnetic nanoparticles onto capillary walls using an external magnetic field to fix them to the inner capillary wall [25]. This research group then extended their initial work to core/shell magnetic nanoparticles, modifying these materials with amino‐ or octadecyl functional groups [26]. Okamoto et al. reported the preparation of a fritless column using magnetic particles with immobilized avidin for chiral separation in 2007 [27]. Liang et al. used BSA‐conjugated graphene oxide‐magnetic nanocomposites for the OT‐CEC separation of tryptophan, threonine, and propranolol enantiomers [28]. In addition to this work, numerous other manuscripts have been published utilizing HSA/BSA immobilization onto various types of stationary phases for enantiomeric separations by CEC [29]. As for the possibility of studying plasma protein–drug interactions, Wang et al. recently presented a novel OT‐ACEC method based on BSA immobilized on polydopamine and metal–organic frameworks to study the interaction of drugs with BSA, specifically three flavonoids and three fluoroquinolones with different affinities for BSA [30].

The pressure‐assisted CEC (PA‐CEC) approach presented here could be particularly useful for studying the binding of drugs to proteins that are either expensive or rare. As an example, Anguizola et al. [31] presented an affinity chromatography study investigating the interaction between sulfonylurea drugs and in vivo glycated HSA, which was isolated from the blood of diabetic patients and immobilized onto an affinity microcolumn. These changes in drug binding could be applied to personalized medicine, also known as precision medicine, to improve healthcare for individual patients, making it smarter, better, and more cost‐effective [32, 33]. Similar studies could also include other affinity pairs.

The approach proposed in this manuscript for studying interactions between plasma proteins and drugs uses a relatively low amount of the protein, compared to ACE modes with free protein. However, the possibility of using fresh protein for individual analyses, as is typically done by other ACE modes, is lost. On the other hand, ACEC columns can be used repeatedly. Furthermore, this eliminates the reduced sensitivity of UV detection due to the presence of protein in the BGE when using msACE.

## 2 Materials and Methods

### 2.1 Materials and Reagents

The chemicals were obtained from the following suppliers: SiMAG‐Amine magnetic maghemite core particles with a silica shell and a 0.75 µm diameter, which were NH_2_‐functionalized, from Chemicell GmbH (Berlin, Germany); a 25% (v/v) solution of glutaraldehyde (GA) from Merck (Darmstadt, Germany), pyridine, BSA; glycine, a Quantipro bicinchoninic acid (BCA) assay kit, 2‐[4‐(2‐hydroxyethyl)piperazin‐1‐yl]ethane‐1‐sulfonic acid (HEPES), NaOH, 1‐ethyl‐3‐(3‐dimethylaminopropyl)carbodiimide (EDC), sulfo‐N‐hydroxysuccinimide (S‐NHS), 2‐(N‐morpholino)ethanesulfonic acid (MES), disodium phosphate, and monosodium phosphate from Sigma‐Aldrich Chemie GmbH (Steinheim, Germany). All chemicals used were of analytical grade or the highest available purity.

The solutions were prepared using deionized water (DW) from a Direct Q 3UV system (Millipore, Milford, MA, USA). The BGE was prepared by adjusting the pH of a 20 mM HEPES solution to 7.4 with 1 M NaOH. MES buffer was prepared by adjusting the pH of a 100 mM solution with 1 M HCl to values of 5 and 6. For the phosphate buffer (PB), 67 mM disodium phosphate solution was adjusted to pH 7.4 with a 67 mM solution of monosodium phosphate.

### 2.2 BSA Immobilization

The immobilization procedure, which was adopted from [34] and then optimized, was finally carried out as follows: iron oxide core magnetic particles, with a diameter of 0.75 µm and NH_2_ functionalized groups on the surface, were stored at 4°C and vortexed thoroughly prior to use. The following activation steps were performed in parallel, taking into account the preparation of particles with immobilized BSA and blank/control particles in which BSA was replaced with glycine.

A 100 µL volume of the particle suspension (50 mg/mL, i.e., about 5 mg of particles) was added to a 2 mL test tube. The particles were first washed with 500 µL DW, followed by three washes with 500 µL of 10 mM pyridine buffer, pH 5.5, as an incubation buffer (IB). The particles' surfaces were then activated with 500 µL of 5% (v/v) GA in DW for 3 hours while on a Bio RS‐24 rotator (Biosan, Riga, Latvia). Afterwards, the activated particles were washed with 500 µL of IB.

The activated particles were incubated for 24 hours on a rotator with 500 µL of 10 mg/mL BSA in IB (5 mg BSA) or 500 µL of 1 M glycine in IB, respectively. The particles were then washed three times with 500 µL of IB. Finally, to block the remaining active groups, the particles were incubated in 500 µL of 1 M glycine in water at pH 8.0 for three hours on a rotator. The aim of incubating the blank/control particles twice with glycine was to ensure that BSA and blank/control particles underwent the same preparatory procedure. The whole procedure is depicted in Figure 1.

**FIGURE 1:**
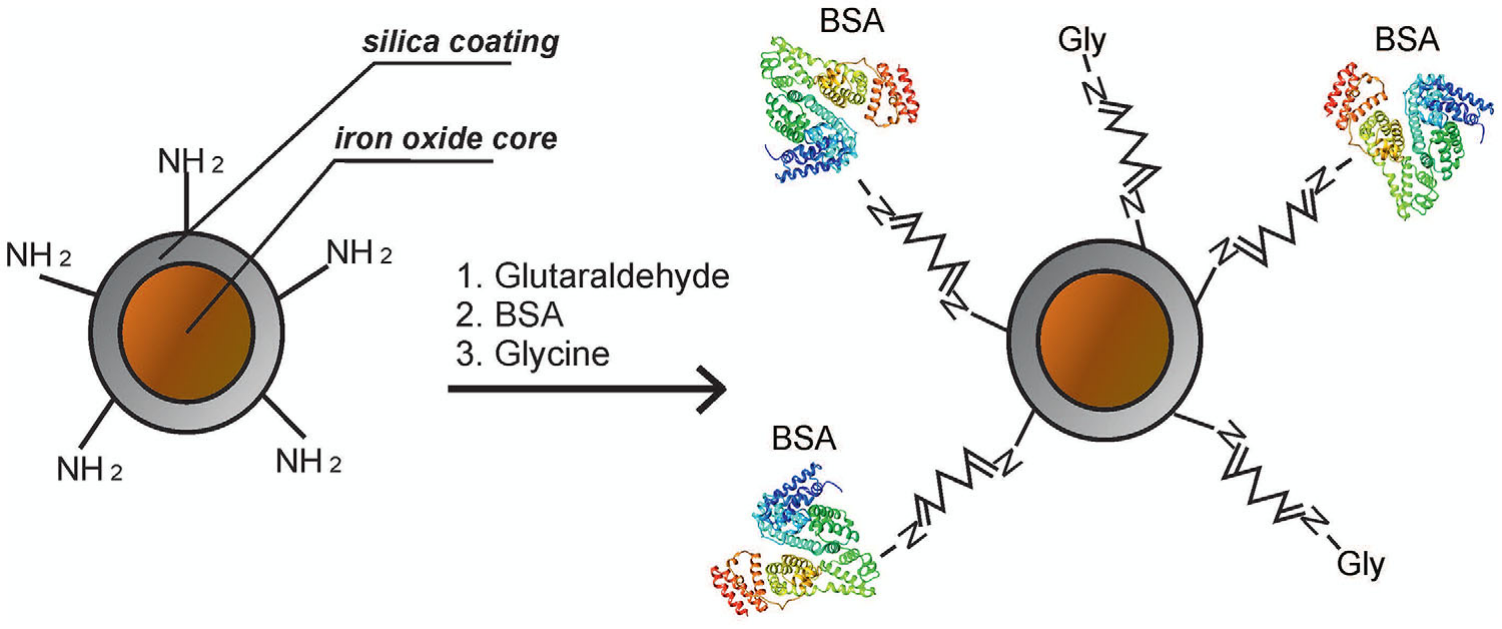
Immobilization of bovine serum albumin (BSA) onto magnetic particles using dialdehyde chemistry. BSA is covalently immobilized onto glutaraldehyde (GA)‐activated NH_2_ groups, and the rest of the active groups are blocked with glycine.

All particle washing steps were performed using a 12 Tube Magnetic Rack magnetic separator (BioLabs, Ipswich, MA, USA), with a 1‐min waiting time for the particles to settle towards the magnet in each step.

All these steps were performed at 25°C. The prepared particles were resuspended in IB to 2 mg/mL or to different concentrations used during optimization, divided into 100 µL aliquots, and stored at 4°C.

The carbodiimide method was tested in a similar way; details are in the supplementary material (SM Carbodiimide immobilization procedure).

### 2.3 Column Preparation

A new capillary with an excess length of 1 cm at the loading end to connect to the syringe was placed in a capillary cartridge in a two‐piece 3D‐printed plastic holder to which 25 pairs of neodymium magnets (4×3 mm, each with a magnetic force of 5 N; Magsy, Fryšták, Czech Republic) were glued in attraction (opposite poles towards each other) as shown in Figure 2. Since individual pairs of magnets could repel each other, they were arranged so that their poles alternated, which resulted in the overall magnetic field generating an attractive force for column formation.

**FIGURE 2:**
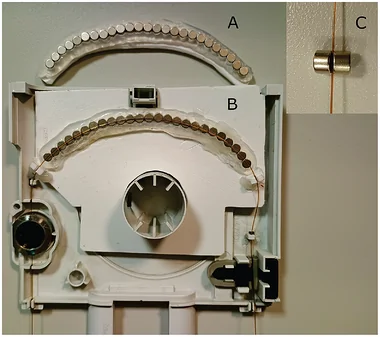
Agilent 7100 CE capillary cassette with home‐made plastic holder with 25 pairs of NdFeB magnets; (A) upper holder part, (B) bottom holder part, and (C) side view of a pair of magnets placed on the capillary.

A bare silica capillary (inner diameter: 75 µm; total length: 34,5 cm; effective length: 26 cm) was positioned in the middle between the pairs of magnets using adhesive tape. It was then preconditioned by rinsing with 1 M NaOH for 15 min, DW for 10 min, and BGE for a further 15 min in a CE instrument. All rinsing steps were performed at a pressure of 950 mbar (95 kPa).

The cartridge was removed from the CE instrument, and a 10 µL pipette tip was attached to the inlet end of the capillary and secured with a hot glue gun (see SM Figure S2). Prior to loading into the capillary, an aliquot of particles was allowed to reach laboratory temperature, after which it was washed three times with BGE using magnetic separation. Immediately before loading, the particle solution was resuspended by vortexing and immediately aspirated into a 100 µL syringe (Hamilton Company, Bonaduz, Switzerland). The particles were then loaded into the capillary using a KDS 100 syringe pump (KD Scientific, Holliston, USA). The loading conditions were as follows: flow rate 200 µL/h, loaded volume 25 µL, and particle concentration 2 mg/mL. All these steps were performed as quickly as possible, as particles tend to sediment inside the syringe, which could affect the repeatability of column preparation and therefore its analytical performance. In this regard, the optimal conditions were chosen as the highest flow rate and total particle mass at which the particles did not leak out, and a concentration that enabled a column to form without clogging the capillary.

### 2.4 Instrumentation

All PA‐CEC experiments were performed on an Agilent 7100 CE system equipped with a diode‐array UV‐Vis detection system (Agilent Technologies, Waldbronn, Germany). Analyses were carried out using a 75 µm I.D., 375 µm O.D. fused silica capillary (34.5 cm total length and 26 cm effective length) from Polymicro Technologies (Phoenix, AZ, USA). The capillary temperature was controlled at 25°C. Data were collected and analyzed using ChemStation software (Agilent Technologies) and Excel 2021 (Microsoft, Redmond, WA, USA).

### 2.5 PA‐CEC Studies

Before each analysis, the column was conditioned with 20 mM HEPES, pH 7.4, as the BGE for 5 min at 950 mbar (95 kPa). Solutions of PBZ standards, dissolved in BGE at concentrations of 25, 50, 100, 150, 200, 300 and 400 µM, containing 0.1% (v/v) acetone as the EOF marker, were electrokinetically injected continuously using a voltage of 15 kV and an additional pressure of +30 mbar (3 kPa) to maintain analysis stability. The separation temperature was controlled at 25°C during all the analysis steps. UV‐Vis data were collected at 265 nm, with a 4 nm bandwidth. Subsequently, after equilibrating the column with a PBZ‐free BGE, the column was fed with the PBZ at different concentrations in BGE for another cycle to obtain a series of breakthrough curves for each PBZ concentration.

### 2.6 Data Processing

The evaluation of frontal PA‐CEC experiments was based on their similarity to frontal analyses (FA) using high‐performance affinity chromatography (HPAC) [35].

In a typical HPAC FA, a sample containing a known total concentration of interacting analyte A [A_0_] is continuously applied to a column containing a fixed amount of immobilized analyte B [B_0_], and the breakthrough elution volume (*V_I_*) is measured. A sample containing the non‐interacting analyte *N_I_* is analyzed in a similar way, and its breakthrough elution volume (*V_NI_*) is measured. The difference between *V_I_* and *V_NI_* was then used to calculate the amount of analyte A (m_Lapp_) that forms a complex with the immobilized analyte B (see Figure 3/SM Figure S1). The substance amount *m_Lapp_* (equal to *n* [mol]) corresponds to the area between the two breakthrough curves and can be calculated by multiplying the difference in their breakthrough elution volumes (*V_I_*_—_ *V_NI_*) by the input ligand concentration [A_0_], as stated in Equation (1).

(1) $$m_{Lapp}=\left[A_{0}\right]\cdot \left(V_{I}-V_{\mathrm{NI}}\right)$$

**FIGURE 3:**
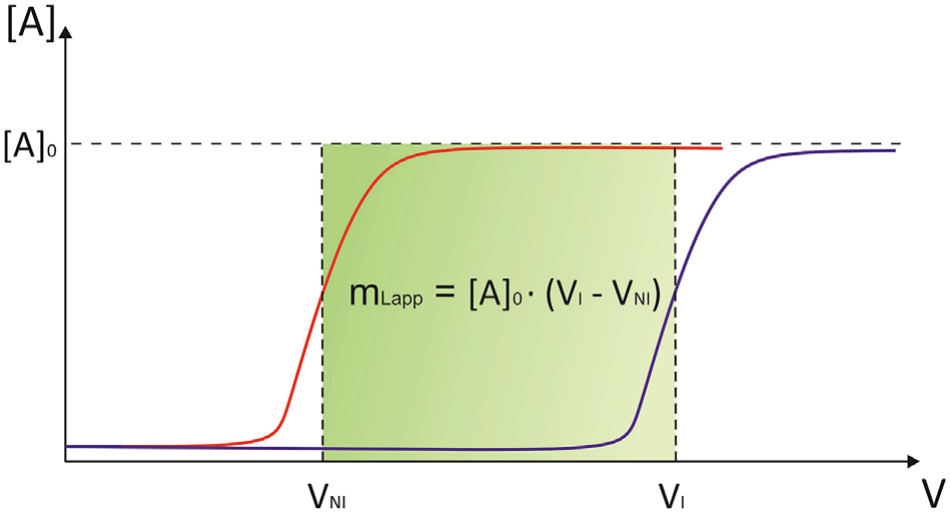
Schematic representation of apparent amount of interacting ligand A (*m_Lapp_*) calculation from breakthrough volumes of noninteracting compound (*V_NI_*) and interacting ligand (*V_I_*), assuming both substances have the same molar extinction coefficient.

To determine the moles of binding sites in the column (*m_L_*) and the *K_b_*, the following equation (Equation (2)), commonly used in frontal affinity chromatography [36, 37], can be applied for the one‐site model:

(2) $$m_{Lapp}=\frac{m_{L}K_{b}\left[A_{0}\right]}{\left(1+K_{b}\left[A_{0}\right]\right)}$$

Taking into account the difference in their basic principles, the theory of frontal HPAC described above has been adopted for the PA‐CEC method presented here. In chromatographic separations, the mobile phase is driven by pressure, with the only separation mechanism being the interaction of the analyte with the stationary phase. In electrochromatographic separations, however, the BGE is driven by the EOF, with the separation mechanism including the interaction with the stationary phase as well as the inherent electrophoretic mobility of the analyte. Therefore, instead of searching for an analyte that does not interact with the stationary phase and at the same time has an identical electrophoretic mobility, which is almost impossible, a blank/control column was used in which no BSA was added during the particle modification process, and the surface was blocked with glycine instead. During analyses on both BSA‐modified and blank/control columns, 0.1% (v/v) acetone was added as an EOF marker to PBZ solutions to address the total flow rate difference between columns. The EOF difference could be attributed mainly to different backpressures and possible different charges introduced by the columns. This approach allowed the PBZ migration change, attributed to affinity, to be analyzed directly. As a result, the correction for the different EOF values of the columns was included in the calculation in both cases.

The breakthrough times of the PBZ and EOF marker in each analysis were determined as the local maximum of the first derivative of the respective breakthrough curve. Using the known effective capillary length and its inner diameter, the EOF velocities were converted into breakthrough volumes, and *m_Lapp_* was calculated for each PBZ concentration according to Equation (3).

(3) $$\;m_{Lapp}=\left(t_{PBZ,\;BSA,corr}-\;t_{PBZ,\;Blank,\;app}\right)\;\cdot \;F_{A}\;\cdot \;\left[A_{0}\right]$$

$t_{PBZ,\;BSA,corr}$ [min] is the breakthrough time of PBZ on the BSA column corrected to the EOF mobility on the blank/control column, $t_{PBZ,\;Blank,app}$ [min] is the breakthrough time at the given concentration of PBZ [A_0_] measured on the blank/control column, respectively; and $F_{A}$ [L/min] is the flow rate of the PBZ zone on the blank/control column.

Details on the data evaluation procedure are provided in the supplementary material (SM Data processing).

## 3 Results and Discussion

### 3.1 Immobilization Chemistry Choice and Optimization

Two different chemical methods, dialdehyde and carbodiimide couplings, which have already been successfully used by our group as described in [34, 38, 39, 40, 41], were tested for the covalent immobilization of BSA onto the surface of magnetic particles. In this context, it should be noted that dialdehyde coupling requires two NH_2_ groups, whereas carbodiimide coupling requires a COOH group and an NH_2_ group, one of which must be present on the relevant protein and the other on the magnetic particles.

GA is the most commonly used reagent for coupling with dialdehydes. Two aldehyde groups separated by an aliphatic chain form a bond between two NH_2_ functional groups present on the magnetic particles and protein, respectively. According to the literature and our previous experience, a two‐step procedure was applied, involving a washing step between particle surface activation and protein addition (see Materials and Methods).

In the carbodiimide coupling reaction, EDC and S‐NHS were employed. First, EDC creates an unstable active group with the COOH group of one partner (the magnetic particles), which can then form a covalent bond with the NH_2_ group of the second partner (the protein). This process can be made more effective by adding S‐NHS, which reacts with the EDC‐activated group to create a more stable active intermediate that then binds covalently to the NH_2_ group of the second partner. This can be done in two different ways: a) a one‐step approach: the protein is added directly after the particles are activated to achieve partial cross‐linking between protein molecules, and b) a two‐step approach: the remaining EDC and S‐NHS are washed away before the protein addition; this procedure was tested in this study to prevent BSA binding sites from being blocked by potential cross‐linking. Both possible set‐ups were tested. The first step of the reaction (activation of the NH_2_ group) took place on the NH_2_ group of the particles or, in the two‐step approach, on the protein's NH_2_ group (COOH‐modified particles). A detailed description of the entire carbodiimide procedure can be found in the supplementary material (SM Bovine serum albumin immobilization).

The amount of immobilized BSA was estimated by analyzing the residual BSA concentration using the BCA assay in the solution after its immobilization. The advantage of GA binding is that the amount of immobilized BSA can be easily determined directly on the particles using the BCA assay, which is not possible with the carbodiimide method. This is due to interference from residual activated groups [42]. An indirect method based on the free, non‐immobilized fraction was finally chosen to ensure that the measurement procedure was the same in both cases.

The binding capacity of immobilized BSA for PBZ was also determined indirectly. This involved direct infusion ESI‐MS analyses of the solutions after the incubation of BSA‐magnetic particles with PBZ and their magnetic separation (both procedures are described in detail in the supplementary material (SM Functional tests of magnetic particles with immobilized BSA). The data are shown in Table 1.

**TABLE 1:** Comparison of different bovine serum albumin (BSA) immobilization chemistries: BSA amount conjugated to magnetic particles (MP) and their binding properties. Buffers: 100 mM MES, pH 5.0 (for MP‐NH_2_); pH 6.0 (for MP‐COOH); 67 mM PB, pH 7.4; and 10 mM pyridine, pH 5.5.

Active groups on MP surface	Chemistry	Buffer/1‐step or 2‐step	µg/mg MP	% bound PBZ	% bound PBZ/µg BSA
─NH_2_	EDC/S‐NHS	MES/1‐step	14.1 ± 1.2	22.30	1.58
─NH_2_	EDC/S‐NHS	PB/1‐step	4.7 ± 1,8	0	0
─NH_2_	GA	Pyridine	40.3 ± 11,3	97.30	2.41
─COOH	EDC/S‐NHS	MES/1‐step	94,4 ± 18.7	95.81	1.01
─COOH	EDC/S‐NHS	PB/1‐step	15.1 ± 1.4	71.06	4.70
─COOH	EDC/S‐NHS	MES/2‐step	100.1 ± 10.2	94.50	0.94
─COOH	EDC/S‐NHS	PB/2‐step	22.6 ± 7.8	44.48	1.97

It should also be emphasized that during the washing and activation steps performed on the magnetic separator, the particles need to be allowed to separate from the solution for a sufficient period of time (at least 1 min) to prevent losses. Additionally, 2 mL tubes should be used for incubation on the rotator to prevent the reaction mixture from getting stuck in smaller tubes' caps and not being mixed properly.

Ultimately, GA coupling was chosen as a compromise, offering a sufficient amount of BSA to be immobilized while maintaining its binding capacity (see Table 1).

The four batches of BSA‐modified particles, used later in ACEC experiments, were prepared by following the procedure described in Materials and Methods—Bovine serum albumin immobilization. The average immobilized amount of BSA was estimated with a BCA kit at 60.6 µg/mg particles.

### 3.2 Column Loading

Prior to being loaded into a pre‐preconditioned capillary already placed in a cassette with the magnet array as presented in Figure 2, the particle suspension was allowed to reach laboratory temperature. The suspension was then thoroughly vortexed and immediately loaded into the capillary. Two approaches of loading syringe and capillary interconnection to reach fast and effective particle transfer were tested: using commercial nanovolume fittings for 360 µm tubing (VICI Jour, Schenkon, Switzerland) and a lab‐made connector formed from a 10 µL pipette tip (see SM Figure S2).

The commercial connector often became clogged with particles when the column was being loaded, which meant that the connector had to be washed and the process had to be repeated. Despite these problems, it was easy to handle. Similarly, the pipette tip connection was relatively straightforward as well, and it never clogged. The pipette tip (common 10 µL) can be attached to the capillary by heating it with a lighter, for example, or a hot glue gun can be used for a stronger connection, as we did.

The optimal conditions for column loading were tested: loading flow rate in the range of 20–600 µL/h, particle concentration in the range of 1–12 mg/mL, and a volume of 5–50 µL. At higher flow rates, the particles collected unevenly (with more particles collected at the far end of the column), and leakage occurred. In addition, high particle concentrations and volume led to clogging of the loading system. In contrast to this, at lower flow rates and suspension concentrations, long loading time led to the sedimentation of particles inside the syringe. The resulting optimization aimed at maximizing the amount of retained particles while maintaining the permeability of the column. Based on microscopic observations, the resulting compromise was a flow rate of 200 µL/h, particle concentration of 2 mg/mL, and loading volume of 25 µL. The columns remained stable under a washing pressure of 950 mbar (95 kPa) and a CE voltage of up to 30 kV. The optimized conditions are summarized in Table 2.

**TABLE 2:** Summarized conditions of column preparation.

Material	Core‐shell magnetic particles 0.75 µm diameter NH_2_ functionalized
Immobilization chemistry	Covalent, GA coupling
Protein	Bovine serum albumin (BSA) 60.6 µg BSA/mg particles
Column loading	Flow rate: 200 µL/h Magnetic particle suspension: 2 mg/mL Injected volume: 25 µL
Magnets holding the column	25 pairs in attraction with opposite poles towards each other (4×3 mm; 5 N each)

### 3.3 Pressure‐assisted Electrochromatography Studies Optimization

Analogous to HPAC, two injection approaches were tested, i.e., zonal and frontal injection analyses (zonal elution and FA according to the typical HPAC nomenclature). In zonal injection, the most common HPAC method for studying affinity interactions, a small amount of the analyte is injected, and the resulting retention factor (k′) depends on its concentration. FA is the second most commonly used variant of HPAC in affinity studies. It involves the continuous addition of a solution containing a known concentration of the solute to be studied into the affinity column. The solute binds to the immobilized ligand, causing saturation, and the amount of solute eluting from the column gradually increases to form a characteristic breakthrough curve. The mean positions of the breakthrough curves can be used to evaluate the association equilibrium constants (see Figure 3).

Firstly, zonal injection was performed in the two injection modes typically utilized for CE analyses: from the short end and from the long end of the capillaries, with effective lengths of 8.5 and 26 cm, respectively. Altria et al. [43] introduced the short‐end injection (SEI) mode to reduce the total analysis time in CE by injecting the sample from the shorter end of the capillary (in this system 8.5 cm) and reversing the polarity of the separation voltage. This mode substantially reduces analysis time and achieves a several‐fold increase in sensitivity [44]. From a separation point of view, it is mainly suitable for simple samples, such as in this case where only PBZ and EOF marker peaks are expected. Due to the cassette's spatial restrictions, only two pairs of magnets could be placed for SEI; 25 pairs were placed around the capillary for long‐end injection, resulting in a long particle bed in the column.

During the subsequent optimization process, the injection parameters of the analytes were examined (various combinations of injection pressures and times in both modes were tested). However, only distorted PBZ peaks were obtained, as shown in the supplementary material (SM Figure S3). On the other hand, the difference in peak shape between the potentially interacting analyte (PBZ) and the non‐interacting analyte (acetone) could imply that an interaction between PBZ and BSA had occurred. Nevertheless, PBZ peak broadening, which was particularly evident at lower concentrations, would lead to significant errors in the peak integration required for the final ACEC experiment evaluation. Based on these findings, the optimization process was continued with continuous frontal sample injection.

Given the analogy between ACEC and HPAC, frontal injection was ultimately tested, in which the analyte is continuously loaded onto the column. The only difference is that electrokinetic injection is used in ACEC instead of pressure injection in HPAC, although an auxiliary pressure of +30 mbar (3 kPa) was used to stabilize the analyses; therefore, the exact terminology for the method should be the above‐mentioned PA‐CEC.

As shown in Figure 4, the continuous electrokinetic injection of PBZ and EOF marker samples led to the formation of analyte plateaus within a reasonable analysis time. Differences in PBZ breakthrough times on BSA and blank/control columns confirmed the interaction between PBZ and BSA. Moreover, the variation in PBZ breakthrough times at different concentrations on the BSA column demonstrated the effectiveness of FA for investigating affinity interactions. Similarly to studies that used HPAC FA [45, 46], it was not necessary to perform any special elution of PBZ from the column between analyses. It was sufficient to simply regenerate the column by allowing BGE to flow through it for 5 min prior to the next analysis, a process which was accompanied by a significant decrease in the absorbance of BGE at 265 nm, returning it to its initial value during this rinse at a pressure of 950 mbar (95 kPa).

**FIGURE 4:**
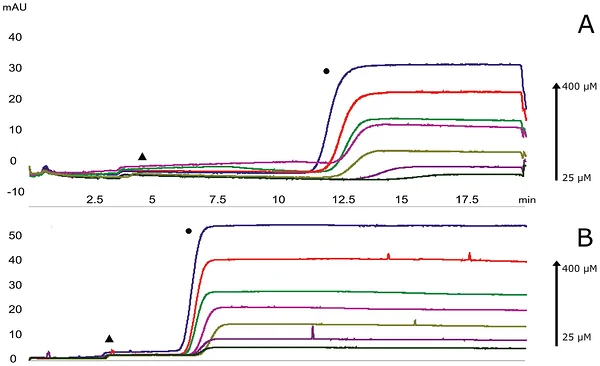
Breakthrough curves of 25, 50, 100, 150, 200, 300, and 400 µM PBZ (•) with 0.1% (v/v) acetone (▲) in BGE; capillary: I.D. 75 µm, total length: 34.5 cm, effective length: 26 cm; injection: continuous electrokinetic (15 kV, +30 mbar (3 kPa)); BGE: 20 mM 2‐[4‐(2‐hydroxyethyl)piperazin‐1‐yl]ethane‐1‐sulfonic acid (HEPES), pH 7.40; Detection: 265 nm; (A) Column with BSA‐modified particles, (B) blank/control column.

This was also supported by the repeatability of the method, as evidenced by the relative standard deviations (RSDs) of the PBZ and acetone breakthrough times. Intracolumn RSD (one column and one analysis series, *n* = 50) of PBZ was 7.81% and of EOF 5.7%. The intercolumn RSD of the breakthrough time (four separate columns, same particles from one immobilization batch) of PBZ at different concentrations (50–400 µM) ranged from 3.0% to 11.0%, and the RSD of the EOF marker (0.1% [v/v] acetone) was 2.3%. Furthermore, EOF mobility, an important factor in data evaluation, typically stabilizes after 10 runs on a new column and remains stable for at least the next 50 analyses.

It should be noted that CEC columns are generally prone to air bubble formation, even after thorough degassing. This can lead to a reduction or even a complete interruption of the electric current. So, several approaches were employed to eliminate this phenomenon. HEPES, a buffer with low conductivity and a pH buffering range close to physiological conditions, was selected as the BGE, and the solution was sonicated thoroughly before use. Although primal tests showed that high voltages of up to 30 kV could be used with this BGE, finally the separation voltage was set to 15 kV just to prevent excessive Joule heating and electric current distortions. Additionally, a pressure of +30 mbar (3 kPa) in the same direction as the EOF was applied to prevent bubble formation while slightly shortening the analysis time without significantly affecting the shape of the peak front.

### 3.4 Determination of BSA‐PBZ Binding Parameters and Data Processing

Based on the results of the optimization experiments, frontal injection analysis was ultimately chosen to determine the PBZ binding parameters. Although FA had previously been employed in CEC for the enrichment of the analyte [47, 48] or the characterization of columns [49, 50], it had never been utilized for interaction studies. In this study, the corresponding experiments involved the continuous electrokinetic injection of samples containing different concentrations of PBZ (25–400 µM) and a constant concentration of the EOF marker (0.1% (v/v) acetone) onto a column containing either magnetic particles modified with BSA or blank/control particles modified with glycine (see Figure 4). The breakthrough times read from the obtained electrochromatograms as the first derivative of the corresponding breakthrough curves are the first variables needed for subsequent evaluation.

The second important variable for evaluating the binding parameters by ACEC is the EOF velocity (see SM Data processing), which was also determined from the aforementioned analyses. As can be seen in Figure 5, the EOF velocity was lower in the analyses performed on the BSA‐modified column than on the blank/control glycine column, both being substantially lower than the EOF on the bare capillary. This effect could be caused by variations in the columns' hydrodynamic resistance combined with the influence of the charged groups of the BSA/glycine and the particle surface.

**FIGURE 5:**
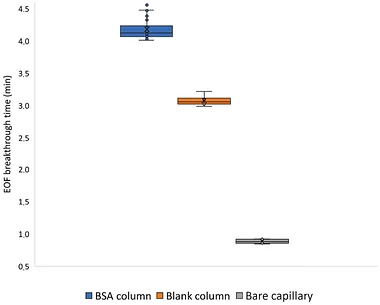
Breakthrough times of electroosmotic flow (EOF) marker (0.1% acetone) on bovine serum albumin (BSA)‐modified column (*n* = 30), blank/control column (*n* = 6) and bare capillary (*n* = 6).

The m_Lapp_ required to saturate the column was calculated for each analyzed PBZ concentration [A₀], under identical conditions for both the BSA‐modified and blank/control columns. The binding parameters *K_b_* and *m_L_* were calculated using Equation (2). The particles used originated from the same batch, which was stored at 4°C. The average values of *K_b_* and *m_L_* of the prepared columns (*n* = 4) were (1.49 ± 0.70) × 10^4^ L/mol and (1.80 ± 0.58) × 10^−11^ mol, respectively. For comparison, the *K_b_* values reported in the literature for the PBZ‐serum albumin interaction determined by different methods are 1.40 × 10^4^ L/mol [51] (fluorescence spectroscopy), 3.57 × 10^4^ L/mol [52] (fluorescence spectroscopy), 1.19 × 10^5^ L/mol [53] (fluorescence quenching), 4.22 × 10^5^ L/mol [54] (isothermal titration microcalorimetry), and 7.60 × 10^5^ L/mol [55] (equilibrium dialysis). The values differ based on the method used. Our data are closer to the lower values of the reported *K_b_* interval. This difference may indicate a lower availability of binding sites, a common disadvantage of immobilization techniques.

### 3.5 Potential of Immobilized Protein and Capillary Reusability

Magnetic particles can easily be released from the capillary by removing the permanent magnets and washing them off with BGE. They can then be captured in a CE vial. The capillary and captured particles can then be reused. While it is relatively simple to reuse the capillary to prepare a column with different interaction partners or for a different type of CE separation, using any standard reconditioning procedure, it is relatively difficult, if not almost impossible, to reuse the released magnetic particles, even after collecting and washing them with a magnetic separator.

The reusability of the capillary was verified on a used capillary after the magnetic particles had been washed out. It was rinsed with NaOH and BGE in the same way as during capillary preconditioning. The migration time of the EOF marker and the electric current both returned to their original values (data not shown).

However, the total number of particles in a single column is low (with a packing volume of 25 µL and a particle concentration of 2 mg/mL used during preparation), making it difficult to prevent potential losses during elution and regeneration. Therefore, this amount of particles would not be sufficient to prepare a new column unless they were collected in larger quantities from multiple columns and regenerated using a suitable washing procedure. Furthermore, the stability of the proteins should be assessed. This was not investigated further in this study, because BSA is a relatively stable protein compared to more complex proteins, such as enzymes or antibodies.

## 4 Conclusions

To our knowledge, this is the first time that a study of affinity drug interactions with BSA as a representative plasma protein has been reported for a column based on magnetic particles by PA‐CEC. Using magnetic particles as the chromatographic bed material offers several advantages, such as easy handling during the chemical activation of their surface and subsequent protein immobilization, and relatively simple column preparation using external permanent magnets. An immobilization method using GA offered several advantages compared to the carbodiimide approach. Namely, a high amount of BSA was immobilized while retaining its ability to bind the model drug PBZ. Furthermore, this chemistry allowed the amount of immobilized protein to be determined directly on the particles using a BCA assay kit. Nevertheless, this possibility was not pursued in this investigation.

A further increase in the required binding capacity, primarily represented by the total amount of immobilized BSA, was achieved by using a long bed of particles retained within the capillary by 25 pairs of permanent magnets in an attractive orientation. This resulted in a longer analysis time of 20 min, including column regeneration, which limits the high‐throughput capabilities of this approach to some extent. However, this is offset by the usefulness of FA for studying weak interactions, like in the case of FA by HPAC.

This approach could be particularly useful for studying drug binding to expensive or rare proteins. The immobilized protein can be used for multiple analyses on a single column. Furthermore, the particles with immobilized protein could be released from the capillary by removing the external magnets and can be regenerated and reused if necessary. However, the particle recovery and the stability of the protein bound to the particles need to be investigated, since the particles tend to adsorb onto labware, and many proteins require great care due to their low stability. It is also worth highlighting the possibility of using the prepared capillary column in capillary array electrophoresis with a higher sample throughput.

## Author Contributions

**Michal Sedlák**: methodology, data curation, investigation, conceptualization, writing – original draft, and writing – review and editing. **Lenka Kohútová**: conceptualization, methodology, validation, writing – original draft, and writing – review and editing. **Marta Pelcová**: conceptualization, methodology, data curation, and writing – review and editing. **Zdeněk Glatz**: conceptualization, methodology, supervision, writing – original draft, and writing – review and editing.

## Ethics Statement

The authors have nothing to report.

## Conflicts of Interest

The authors declare no conflicts of interest.

## Declaration of Generative Artificial Intelligence and Artificial Intelligence‐assisted Technologies in the Writing Process

The authors confirm that generative Artificial Intelligence (AI) or AI‐assisted technologies were not used to produce scientific insights, to analyze or interpret data, or to draw conclusions.

## Supporting information




**Supporting File**: jssc70570‐sup‐0001‐SuppMat.docx.

## Data Availability

The data that support the findings of this study are available from the corresponding author upon reasonable request.
