# Supplementary material for: Magnetic Microparticles With Immobilized Bovine Serum Albumin as a Stationary Phase for Affinity Studies Using Pressure‐Assisted Capillary Electrochromatography. Proof of Concept
Source: J Sep Sci. 2026 Nov 9;49(11):e70570. doi: 10.1002/jssc.70570 (PMC13748917; doi:10.1002/jssc.70570)
Supplement: Supplementary file 1 [file JSSC-49-e70570-s001.docx]

**SUPPLEMENTARY MATERIAL**

**Magnetic microparticles with immobilized bovine serum albumin as a potential stationary phase for affinity studies using pressure-assisted capillary electrochromatography. Proof of concept.**

Michal Sedlák, Lenka Kohútová, Marta Pelcová, Zdeněk Glatz,

Department of Biochemistry, Faculty of Science, Masaryk University,

Kamenice 5, 625 00 Brno, Czech Republic

Corresponding author:

Prof. RNDr. Zdeněk Glatz, CSc

Department of Biochemistry, Faculty of Science, Masaryk University,

Kamenice 5, 625 00 Brno, Czech Republic

E mail: glatz@chemi.muni.cz Tel: + 420 549 49 3957 Fax: + 420 549 49 2690

**Table of contents**

**Data processing…………………………………………………………………………S3**

**Bovine serum albumin immobilization………..………………………..…………..S5**

**Glutaraldehyde immobilization procedure….………………….…………S5**

**Carbodiimide immobilization procedure………….………………………S7**

**Functional tests of magnetic particles with immobilized BSA………….……...S9**

**The amount of immobilized BSA…………………………….…………..…S9**

**Binding capacity of magnetic particles with immobilized BSA……….S9**

**Syringe-to-capillary connection……………………………………………………...S11**

**Zonal injection tests on BSA column……………………………………………….S11**

**References……………………………………………………………………………….S12**

**Data processing**

The evaluation of frontal ACEC experiments was based on their similarity to frontal analyses using high-performance affinity chromatography (HPAC). In a typical HPAC frontal analysis, a sample containing a known concentration of pure interacting analyte A [A₀] is continuously applied to a column containing a fixed amount of immobilized analyte B [B₀], and the breakthrough elution volume (V_i_) is measured. A sample containing the non-interacting analyte N_I_ is analyzed in a similar way, and its breakthrough elution volume (V_NI_) is measured to determine the amount of analyte A that forms a complex with the immobilized analyte B (see Fig. S1). This amount (m_Lapp_) corresponds to the area between the two breakthrough curves and can be calculated by multiplying the difference in their breakthrough elution volumes (V_I_ - V_NI_) by the input ligand concentration [A_0_].

**Figure S1** Schematic representation of apparent moles of interacting ligand A (m_Lapp_) calculation from breakthrough volumes of noninteracting compound (V_NI_) and interacting ligand (V_I_).

To determine the substance amount of binding sites in the column (m_L_) and the equilibrium binding constant (K_b_), the following equation, commonly used in frontal HPAC [1, 2] for the one-site model, can be applied:

$m_{Lapp}=\frac{m_{L}K_{b}\left[ A0 \right]}{\left( 1+K_{b}\left[ A0 \right] \right)}$	(1)

Parameters $m_{L}$ and $K_{b}$ are calculated from its linearized form (see equation 2) as following $m_{L}$ *= 1 / intercept*; $K_{b}$ *= intercept / slope :*

$\frac{1}{m_{Lapp}}=\frac{1}{K_{b}m_{L}\left[ A0 \right]}+\frac{1}{m_{L}}$	(2)

In view of the difference in their basic principles, the BGE driven by the EOF in electrochromatographic separations in contrast with the mobile phase driven by pressure in chromatographic separations, the theory of frontal HPAC described above has been adopted for ACEC, with the breakthrough times and mobilities used for calculation of breakthrough volumes.

Apparent substance amount of ligand A, $m_{Lapp}$ [mol], needed to saturate the column at a given ligand concentration [A_0_] [mol/ dm^3^] was calculated using the following equation 3:

$m_{Lapp}=\left[ A_{0} \right]\cdot(V_{I}- V_{NI})$	(3)

($V_{I}$ - $V_{NI}$) [dm^3^] correspond to volume difference of PBZ zone needed to saturate the column at different ligand concentrations (to achieve breakthrough time) on BSA column and blank column and is calculated as $F_{A}$ [dm^3^/min], PBZ apparent flow rate on blank column, multiplied by difference between PBZ breakthrough time on BSA column with EOF correction and PBZ breakthrough time on blank column as presented in equation 4:

$\left( V_{I}- V_{NI} \right)= F_{A} \cdot(t_{PBZ, BSA,corr}- t_{PBZ, Blank, app})$	(4)

PBZ zone flow rate at different concentrations of PBZ on blank column$F_{A}$ [dm^3^/min] is calculated as effective capillary volume $V_{eff}$ [dm^3^] divided by $t_{PBZ, Blank, app}$ [min], PBZ breakthrough time at different ligand concentrations on blank column (equation 5)

$F_{PBZ,Blank} = \frac{V_{eff}}{t_{PBZ, Blank, app}}$	(5)

$V_{eff}$ is capillary volume from inlet to detector (to breakthrough time detection point), calculated as $S_{cap}$ [dm^2^], capillary inner cross-section area, multiplied by $l_{eff}$ [dm], effective length expressed in equation 6

$V_{eff} = S_{cap}\cdot l_{eff}$	(6)

Corrected PBZ time on BSA column (correction to blank EOF) $t_{PBZ, BSA,corr}$ [min] is calculated from following equation 7:

$t_{PBZ, BSA,corr}= \frac{l_{tot} \cdot l_{eff}}{\left( \mu_{PBZ, BSA, eff}+ \mu_{EOF, Blank} \right)\cdot U}$	(7)

Where $l_{tot}$ and $l_{eff}$ [dm] are total and effective capillary lengths, $\mu_{PBZ, BSA, eff}$ and $\mu_{EOF, Blank}$ [dm^2^.V^-1^.min^-1^] are PBZ effective mobility on BSA column and EOF mobility on blank column at different PBZ concentrations, respectively, $U$ is applied voltage.

**Bovine serum albumin immobilization**

The SIMAG iron oxide core magnetic particles (Chemicell GmbH, Berlin, Germany), a diameter of 0.75 µm with NH_2_ or COOH functionalized groups on the surface, were stored at 4 °C and vortexed thoroughly prior to use.

All particle washing steps were performed using a 12 Tube Magnetic Rack magnetic separator (BioLabs, Ipswich, MA, USA), with a 1-minute waiting time for the particles to settle towards the magnet in each step.

***Glutaraldehyde immobilization procedure***

BSA was immobilized onto surface of SiMAG -NH_2_ particles. The immobilization buffer (IB) was 10 mM pyridine buffer, pH 5.5

100 µL of the 50 mg/mL particle suspension (5 mg of particles) was added to a 2 mL test tube. The particles were first washed with 500 µL deionized water (DW), followed by three washes with 500 µL of IB. The particles' surfaces were then activated with 500 µL of 5% (v/v) glutaraldehyde in DW for 3 hours while on a Bio RS-24 rotator (Biosan, Riga, Latvia). Afterwards, the activated particles were washed with 500 µL of IB. The activated particles were then incubated for 2 hours at room temperature on a rotator with 500 µL of 10 mg/mL BSA in IB (5 mg BSA).

After the incubation and particle separation, the free solution was pipetted out and stored for further measurement (see below). The particles with immobilized BSA were washed three times with 500 µLof IB and stored in 1 ml of this solution in a refrigerator at 4 °C.

The described immobilization procedure was used only for coupling chemistry optimization, the final method of BSA immobilization onto magnetic particles used for column preparation differs in some steps due to later optimization of the process and is described in detail in the article (**Bovine serum albumin immobilization**)

***Carbodiimide immobilization procedure***

Two-step method

BSA was immobilized onto surface of SiMAG-COOH particles. The IB consisted of 100 mM MES, pH 6 or 67 mM phosphate buffer, pH 7.4

100 µL of the 50 mg/mL particle suspension (5 mg of particles) was added to a 2 mL test tube. The particles were washed with 500 µL of DW, followed by three washes with 500 µL of IB. After the final washing step, 500 µL of 25 mM EDC and 6.25 mM S-NHS in DW was added to the test tube, and the solution was incubated for 10 minutes at laboratory temperature on a rotator. Afterwards, the activated particles were washed with 500 µL of IB. The activated particles were then incubated for 2 hours at room temperature on a rotator with 500 µL of 10 mg/mL BSA in IB (5 mg BSA).

After the incubation and particle separation, the free solution was pipetted out and stored for further measurement (see below). The particles with immobilized BSA were washed three times with 500 µL of IB and stored in 1 mL of this solution in a refrigerator at 4 °C.

One-step method

BSA was immobilized onto surface of SiMAG-NH₂ (MP-NH₂) or SiMAG-COOH (MP-COOH) particles. In contrast with the two-step method, the removal of the activation reagents from the reaction mixture prior to the addition of the diluted BSA solution was omitted. IB consisted of 100 mM MES, (pH 5 for MP-NH₂ and pH 6 for MP-COOH) or 67 mM phosphate buffer, pH 7.4

100 µL of the 50 mg/mL particle suspension (5 mg of particles) was added to a 2 mL test tube. The particles were washed with 500 µL DW and three times with 500 µL IB. After the final washing step, 500 µL of 25 mM EDC activation solution or 25 mM EDC and 6.25 mM NHS solution was added to the tube, and the resulting solution was incubated on a rotator for 10 minutes at room temperature. In the next step, 500 µL of a 10 mg/mL BSA (5 mg BSA) solution in IB was added. The mixture was thoroughly mixed on a vortex and incubated on a rotator for 2 hours at room temperature.

After the incubation and particle separation, the free solution was pipetted out and stored for further measurement (see below). The particles with immobilized BSA were washed three times with 500 µL of IB and stored in 1 mL of this solution in a refrigerator at 4 °C.

**Functional tests of magnetic particles with immobilized BSA**

***The amount of immobilized BSA***

The amount of immobilized BSA was estimated by analyzing the residual BSA concentration using the BCA assay (QuantiPro BCA Assay Kit, Sigma-Aldrich Chemie GmbH, Steinheim Germany) in the solution after its immobilization on magnetic particles and their removal on a magnetic separator. All steps were performed according to the manufacturer’s protocol.

2.5 µL of each solution obtained after the last immobilization step was diluted 400-times with respective IB into final 1 mL volume, each sample in duplicates. BSA calibration solutions were prepared by diluting 1 mg/mL BSA standard solution with IB to concentrations 0, 0.5, 5, 10 ,20 and 30 µg/mL. BCA working reagent was prepared by mixing QA, QB and QC (company protocol) reagents in 25:25:1 ratio. QA (buffer solution; undisclosed concentration of sodium tartrate, Na_2_CO_3,_ NaHCO_3_ and NaOH, pH 11.25) and QB (4% (w/v) bicinchoninic acid, pH 8.5) solutions were thoroughly mixed and after that combined with QC (4% (w/v) CuSO_4_) solution until uniform in colour.

1 mL of BCA working BCA reagent was then added to 1 mL of the samples or calibration solutions, thoroughly mixed and incubated at 60 °C for 1 hour. After that, incubation tubes were rapidly cooled down to 4 °C to terminate the reaction and absorbance at 562 nm was recorded on spectrophotometer (Helios Gamma UV-Vis, Thermo Scientific, USA).

***Binding capacity of magnetic particles with immobilized BSA -*** ***Direct infusion ESI-MS analyses***

2.5 mg of BSA-modified particles were incubated with 300 µL of 2 µM PBZ in 10 mM ammonium acetate buffer, pH 7.4. After 10 minutes incubation and magnetic particle separation, 200 µL of resulting solution was diluted in 1:1 ratio with the ammonium acetate buffer, pH 7.4. Calibration samples were prepared in concentration range from 25 nM to 1.2 µM PBZ in the same buffer.

The PBZ samples and calibration solutions were analyzed by direct infusion ESI-MS analysis. A 20 µL of the sample was injected by A Dionex UltiMate3000 pump (Waltham, MA, USA) with degasser into a maXis impact qTOF (Bruker Daltonics, Bremen, Germany) with ESI source. Mobile phase consisted of 20/80 (v/v) mixture of 10 mM ammonium acetate buffer, pH 7.4 and acetonitrile with flow rate 0.4 mL/min. ESI voltage was set to 3 kV, drying gas flow rate 1.5 L/min and temperature 250 °C. Detection m/z was set to 309 for PBZ precursor ion.

**Syringe-to-capillary connection**

**Figure S2** Tested connection options between a 100 µL syringe and a bare silica capillary: A) commercial connector, B) 10 µL pipette tip secured to the capillary with glue.

**Zonal injection tests on BSA column**

**Figure S3** Representative zonal injection electropherogram at 265 nm. Capillary: I.D. 75 µm, total length: 34.5 cm, effective l.: 26 cm; injection: 50 mbar (5 kPa), 20 s; BGE: 20 mM HEPES, pH 7.40. Sample: PBZ concentration 400 µM (blue line) and 100 µM (red line), both with 0.5% (v/v) acetone.

**References**

[1] K. Kasai, Frontal affinity chromatography: An excellent method of analyzing weak biomolecular interactions based on a unique principle, Biochimica Et Biophysica Acta-General Subjects 1865(1) (2021) 129761. <https://doi.org/10.1016/j.bbagen.2020.129761>.

[2] D.S. Hage, High-performance affinity chromatography: a powerful tool for studying serum protein binding, Journal of Chromatography B 768(1) (2002) 3-30. <https://doi.org/10.1016/S0378-4347(01)00482-0>.
